# On the Treatment of Nursing Sore Mouth

**Published:** 1848-09

**Authors:** 


*On the Treatment of Nursing Sore Mouth.* By Dr. Ely, of Rochester, New York.

The appearance of several articles in the journals, within a comparatively recent period, on the disease familiarly known as "nursing sore mouth," naturally begets the presumption that it is becoming more prevalent and troublesome, and that anything relating to its treatment might not be unacceptable to the profession.

It is the impression with many that this is a new disease, and that nursing and pregnant women are exclusively liable to it. Such is not, however, the universal opinion of the profession. We are informed of its occurrence in other countries, and that a similar affection is found in the male sex. These points are not material for us to decide. We have thought, however, in comparing the description given of "chronic thrush," with that given by some writers of "nursing sore mouth," that if the terms were changed, the description of the disease we are considering would be improved.

A brief history of the complaint will suffice. It is generally attendant on pregnancy or lactation. Those whose blood is impoverished, or whose digestive organs are in a bad state, are especially liable to it. It is not a necessary attendant on anæmia—the worst cases of which may exist without it. Its duration is indefinite; but the change in the system which occurs on the "drying up of the milk," usually puts a stop to it. Burning in the stomach is often the first symptom—then a scalded, hot sensation in the mouth; the mucous membrane is red in patches or throughout—sometimes looks as if stained, simply, especially on the tongue; pimples and ulcers make their appearance, and become foci of inflammation; they frequently occur under the tongue, and between the lips or cheeks and the teeth. The stomach may now be relieved; but when the mouth improves, *its* irritation appears to be renewed, the dyspeptic symptoms varying in different cases, when on the occurrence, perhaps, of diarrhœa, a temporary relief is gained in respect to the stomach and mouth. The disease has its acute and chronic stages, the former being often attended with febrile symptoms. A diarrhœa in the chronic form of the complaint is one of the most troublesome symptoms. It occurs both during pregnancy and lactation, and in the latter state may frustrate our efforts to cure the disease without weaning the child. The exhaustion caused by diarrhœa may prove fatal; sometimes it terminates by the supervention of pulmonary symptoms. A majority of cases recover; some are very protracted, and whatever relief may be gained by treatment, unless the system can be brought into a healthy state, it is exceedingly liable to re-appear.

I have not deemed it desirable to prolong this article by introducing any matter relative to the topography of the disease, its dependence on locality, climate, &c., and kindred questions, and for the same reason forbear to speculate on its pathological characters. It only remains, then, to speak of the treatment.

Not having kept any written data, I regret my inability to furnish precise statistical information of the reults of treatment. I am quite certain, however, that "our experience" has been different from that of a distinguished physician who has lately written on the subject, who has found, "that in a large proportion of cases weaning becomes necessary." (Dr. Flint, in Buffalo Medical Journal, Feb., 1848.) We admit this alternative only in cases of much exhaustion, or where diarrhœa exists to severe or dangerous extent.

Our practice (it may be proper to state) has been, for the last ten years, in an "infected region." About eight years ago the disease was fast becoming an opprobrium medicorum. It was about this time that the paper of Dr. Backus; which has become a standard essay, was written, in which he says that "latterly some cases have resisted this and every other mode of treatment, and I have had to wean the child, when the disease was cured at once and did not return. The allusion is to a compound pill of aloes, rhubarb, carb. iron and ipecac, and astringent and other washes for the mouth. At this time, also, it was announced that a neighboring physician had accidentally discovered *the* specific; which proved to be powdered cubebs, in connection with bismuth, gum Arabic, &c. For the burning in the stomach the cubebs was serviceable, but the most chronic cases resisted the treatment. In March, 1841, a case occurred to me, in which delivery of twins was followed by alarming hemorrhage; this was succeeded by re-action that sometimes attends loss of blood, during which the sore mouth made its appearance, preceded by the burning in the stomach. The ulcers were in the most sensitive part, under the tongue. All motion of the tongue was very painful, and swallowing and speaking were nearly impracticable. The treatment was that commonly supposed to be indicated, and among the local remedies nitrate of silver was employed in substance, and creosote also was freely applied. The case went on unrelieved, although the anæmic symptoms were yielding to appropriate remedies. Believing that some new remedy would be necessary, I was speculating on the probable effects of corrosive sublimate, but determined to make a trial first of the iodo-hydrargyrate of potash. A wash was tried, of sufficient strength to cause some tingling and pain on being held in the mouth, which *at once* relieved the stiffness and soreness of the tongue. A repetition of the remedy a few times changed the diseased action, and the patient had a permanent recovery. I immediately commenced using the medicine internally and locally, and was pleased to find that it might be so managed in almost every case, either by using it alone or in combination with tonics, as to afford a favorable result. I soon found, especially in chronic cases, that when taken into the stomach the mouth did not seem to require its topical use. I have continued to employ it to the present time, and with no abatement of confidence in its efficacy.

During the most active state of the buccal inflamation, it has displayed irritant effects which have contradicted its use; an over-dose on an empty stomach will nauseate; and in some cases I have found more benefit from ferruginous remedies. But notwithstanding these exceptions, it cures the disease with more certainty than any and all other remedies—and its non-salivant property renders it safe as a mercurial alterative. The solution I have used is made by dissolving ten grains each of iodide of potassium and red iodide of mercury in one ounce of rain water—dose, four to six drops three times a day. A minute quantity of this solution, applied to a diseased follicle or pimple, disposes it to heal; and if used as a wash, 6 drops may be added to one or two ounces of water.

In cases where the medicine does not agree, or where a tonic seems preferable, I have used a mixture of iodide of iron, Huxham's tincture and syrup, and a mouth wash of sulphate of copper, rose water and honey.

Where the case is attended with much or protracted *burning* in the stomach, I should prescribe tinct. muriate of iron, and saturated alcoholic tincture of cubebs, in equal proportions, fifteen to thirty drops three times a-day.

It is to be understood, in connection with the above, that no permanent immunity can be obtained from this disease, nor 'can even temporary benefit be rationally expected from the best treatment, without a skilful management of the whole case. Thls I trust will be considered before any one concludes that the medicine will not bear its recommendation.

ROCHESTER, N. Y., July 29, 1848. W. W. ELY.

[*Boston Medical and Surgical Journal.*

